# Towards the incorporation of tipping elements in global climate risk management: probability and potential impacts of passing a threshold

**DOI:** 10.1007/s11625-018-0536-7

**Published:** 2018-02-03

**Authors:** Yoshihiko Iseri, Sayaka Yoshikawa, Masashi Kiguchi, Ryunosuke Tawatari, Shinjiro Kanae, Taikan Oki

**Affiliations:** 1 Department of Civil and Environmental Engineering, School of Environment and Society, Tokyo Institute of Technology, 2-12-1, O-okayama, Meguro, Tokyo, 1528552 Japan; 2 Hydrologic Research Laboratory, Department of Civil and Environmental Engineering, University of California, Davis, One Shields Avenue, Davis, CA 95616 USA; 3 Institute of Industrial Science, The University of Tokyo, 4-6-1, Komaba, Meguro, Tokyo, 1538505 Japan; 4 United Nations University, 5-53-70, Jingumae, Shibuya, Tokyo, 1508925 Japan

**Keywords:** Arctic summer sea-ice, Climate change, Greenland ice sheet, Sea level rise, Tipping elements, Threshold temperatures

## Abstract

Evidence suggests that several elements (i.e., subsystems) of the Earth’s climate system could tip into a qualitatively different state due to on-going and future anthropogenically induced climate change. Risks associated with tipping could form a component of critical climate risks, and their consideration should be indispensable in decision-making. However, there is lack of scientific knowledge about the risks associated with tipping elements, inhibiting their incorporation into comprehensive risk assessments of climate change (i.e., assessments of impact, adaptation, and mitigation with uncertainty). Using two major tipping elements (Arctic summer sea-ice loss and Greenland ice-sheet melting) as examples, this study attempted to address this lack of knowledge by conducting several calculations under various policy choices based on target temperature, including (i) the probability of passing a threshold temperature in this century and (ii) the potential impact of passing a threshold temperature on a millennial timescale beyond this century. The first theme of this study [Item (i) above] suggested that probability of exceeding the threshold within this century is 24.8% for the Greenland ice sheet and 2.7% for Arctic summer sea ice under a 1.5 °C temperature goal. However, it should be noted that the estimated probabilities of exceeding the threshold are largely dependent on the specification of the probability density function and key assumptions. With regard to the second theme of this study [Item (ii) above], estimation of the potential global coastal exposure using the estimated sea level exhibited a significant gap between scenarios not exceeding the threshold (1.5 °C target) and those exceeding the threshold.

## Introduction

Tipping elements (TEs), as introduced by Lenton et al. (2008) and described as abrupt or irreversible changes in the Intergovernmental Panel on Climate Change (IPCC) Fifth Assessment Report (IPCC 2013) with slightly different implications, are elements (i.e., subsystems) within the earth’s climate system that could pass critical thresholds, resulting in the destabilization, destruction, critical damage, or transmutation of the major subsystems of the climate system (Lenton et al. 2008; Schellnhuber et al. 2016). Among the TEs that could be triggered within this century, Arctic summer sea-ice loss and Greenland ice sheet melting are two major TEs that could have large impacts on a global scale. For example, melting of the Greenland ice-sheet could result in a sea level rise (SLR) of more than 7 m (Lenton et al. 2008), although this transition will likely occur on a timescale of several hundreds of years to a millennium. Meanwhile, the disappearance of Arctic summer sea-ice is expected to have a great impact on humans and ecosystems in the Arctic region (Cohen 2014), and the transition could occur in less than 10 years (Lenton et al. 2008). In addition to their potential considerable impacts, these TEs could pass their critical threshold (i.e., thresholds of global mean temperature) within this century, even if the Paris Agreement goal of an “increase in the global average temperature (well) below 2 °C above preindustrial levels” is achieved (Schellnhuber et al. 2016).

The Integrated Climate Assessment—Risks, Uncertainties and Society (ICA-RUS) was a 5-year (2012–2016) research project that had the aim of developing and proposing strategies for global climate risk management, from which several papers, including this paper, have been submitted to the special feature of this journal. Although many of the research components of ICA-RUS focused on the risks and impacts that could appear within this century, and mitigation options that could be implemented in this century, the choice of strategies could lead to drastically different implications with regard to TEs (ICA-RUS Report 2015). For example, if the threshold of a TE is only 1.0 °C, it will inevitably be passed, regardless of which strategy is chosen. However, if the threshold is 2.0 °C, the strategic choice will greatly affect the likelihood of the threshold being passed (ICA-RUS Report 2015).

To consider TEs when developing and proposing strategies (Emori et al. 2018 in this special feature), which are primarily outcomes of integrated assessment models (IAMs) of climate change, there are at least two topics that should be further investigated beyond the literature that was available when ICA-RUS was initiated: the uncertainty surrounding TEs and their threshold temperatures and the consequences of passing threshold temperatures.

The aim of this paper in this special issue was to investigate these two topics based on specific examples of TEs. Based on an investigation of two major TEs (Arctic summer sea-ice loss and Greenland ice sheet melting), we assess (i) the probability of passing thresholds within this century both for Arctic summer sea-ice loss and Greenland ice sheet melting, and (ii) the potential impact of Greenland ice sheet melting beyond the threshold on a millennium timescale. In addition, this investigation provides estimations of the potential impact and probability of exceeding thresholds performed under consistent scenarios using the IAM simulations from ICA-RUS. The ultimate goal of this research was to support the incorporation of TEs and their threshold temperatures into strategies for the global-scale management of climate change risks, which could be realized through the incorporation of TEs and their thresholds into IAMs in a technical sense. Although we have not reached this ultimate goal, the analyses presented herein represent an indispensable preliminary step toward this goal.

The structure of this paper is as follows. The next section focuses on the two investigated TEs. We explain the details of our framework to estimate the probability of exceeding the threshold temperatures of TEs. Then, we apply the proposed framework to two TEs and discuss the results. Following this, we focus on the special case that has the highest probability of occurrence within our framework, and perform a millennial-scale estimation of sea level rise and its consequences considering the threshold behavior of the Greenland ice sheet.

## Probability of exceeding threshold temperatures in the current century

Exceeding threshold temperatures could have catastrophic impacts. However, existing studies on the impacts of temperature increase seldom consider the threshold behavior of climate systems in their assessments. Difficulties in considering climate system thresholds in impact assessments arise from uncertainties associated with the thresholds, including the possible range of threshold temperatures. However, a probabilistic approach can be used to cope with these uncertainties. Several studies using IAMs have evaluated climate impacts while considering the probability of crossing threshold temperatures (e.g., Hope 2006, 2011; Cai et al. 2015; Lontzek et al. 2015). Lontzek et al. (2015) introduced a threshold module into a stochastic version of the dynamic stochastic integrated climate and economy model, which is an IAM that has been widely used for evaluating the economic impacts of climate change (Watkiss 2011). The module’s parameter to set the probability of crossing the threshold temperature was determined by referring to the study of Kriegler et al. (2009), which used expert elicitations from 43 experts. Meanwhile, Screen and Williamson (2017) assessed the probability of an ice-free state Arctic sea focusing on the temperature targets in the Paris Agreement. These studies provide important insights into the probability of exceeding the thresholds of the climate system. However a framework to estimate the probability of exceeding various thresholds has not yet been established, whereas the probabilities estimated using such a framework can be incorporated into IAMs and the importance of incorporating thresholds has previously been discussed (Lenton and Ciscar 2013).

Here, we present a framework to execute a probabilistic assessment of exceeding the threshold of the climate system focusing on two TEs, the Greenland ice sheet and Arctic summer sea-ice. Within our framework, uncertainty in the threshold temperatures is represented by probability density functions (PDFs). The parameters of the PDFs are determined based on a literature review, enabling us to obtain the PDF for each TE separately. In this study, temperature increase represents the temperature anomaly with respect to preindustrial levels.

### Methodology used to estimate the probability of exceeding the threshold temperatures

**Fig. 1:**
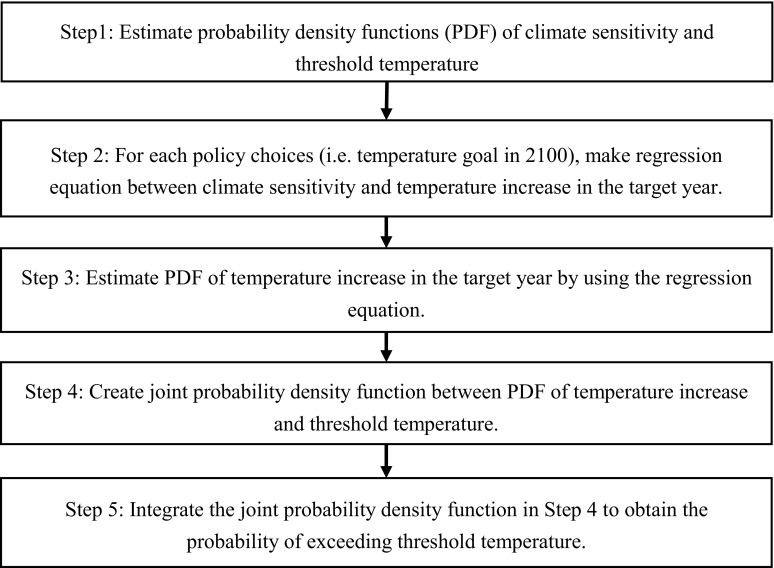
Flow chart of a method for estimating the probability of exceeding the threshold temperature by a target year under different policy choices

Figure 1 presents the algorithm used to assess the probability of exceeding the threshold temperatures of the TEs. In this algorithm, the uncertainties of the global mean temperature increase and the threshold temperature of the TEs are represented by PDFs. For the two major TEs, we set the PDF and threshold temperature of the two TEs based on the following rationale.

First, the threshold of Greenland ice-sheet melting is defined as the temperature increase at which the surface mass balance of the Greenland ice sheet becomes negative. It should be noted that it may take hundreds of years to reach a negative mass balance after the temperature increases to the threshold. In the AR5 (IPCC 2013), the threshold temperature of the Greenland ice sheet is given as greater than 1 °C (low confidence) and lower than 4 °C (medium confidence). These statements suggest that we can be relatively confident that the threshold of Greenland ice sheet melting is below 4 °C, and also implies that the threshold might be lower, and closer to 1 than 4 °C. Robinson et al. (2012) estimated the threshold temperature to be 1.6 °C with a 95% confidence interval of 0.8–3.2 °C. In addition to the above studies, Leverman et al. (2013) discussed the threshold of abrupt Greenland ice sheet loss to be between 0.8 and 2.2 °C (90% credible interval). Based on these values, we employed a log-normal distribution for the threshold temperature of Greenland ice-sheet melting using 1.6 °C as the best estimate and 1.0–4.0 °C as the 95% confidence interval., This setting of the PDF is consistent with our assumption of a threshold around 1.6 °C with a right-skewed distribution. Second, the threshold of Arctic summer sea-ice loss is the temperature at which the Arctic Ocean is in a nearly ice-free in September, and was determined as follows. Several models have estimated that a largely ice-free Arctic sea in summer would begin around 2 °C above the present temperature (Lenton 2012). Massonnet et al. (2012) identified a Coupled Model Intercomparison Project Phase 5 (CMIP5) subset to represent September Arctic sea ice extent, indicating an increase of 1.6–2.1 °C (mean 1.9 °C) from the present period (2.2–2.7 °C and mean 2.5 °C from preindustrial levels) as the annual mean global surface temperature threshold for a nearly ice-free state of the Arctic Ocean in September. Therefore, we set the PDF for the threshold temperature of Arctic summer sea ice of 2.45 °C as the mean and 2.2–2.7 °C as the 68% confidence interval. The mean of 2.45 °C was almost consistent with a mean value of 2.5 °C in the CMIP 5 subset presented in Fig. 12.31e of AR5 (IPCC 2013), and did not conflict with the best estimate of ~ 2.0 °C (above present) given by Mahlstein and Knutti (2012). Meanwhile, setting 2.2–2.7 °C as the 68% confidence interval (1.95–2.95 °C as the 95% confidence interval) enabled the possibility of the threshold falling in the range beyond of 2.2–2.7 °C, which should be considered because of the large variabilities of the thresholds in the models presented in Figs. 12.30 and 12.31 of the AR5 (IPCC 2013).

To estimate the probability of exceeding the thresholds by the end of this century, we used a set of global mean temperature increase projections conducted as IAM simulations in ICA-RUS. The IAM simulations were performed for several policy choices based on the target temperature at the end of this century. Details of the IAM simulations can be found in Su et al. (2017), who computed four sets of future emission pathways, including T15, T20, and T25. Each emission pathway corresponds to a temperature pathway. These pathways each correspond to a policy target that limits the temperature increase in 2100 to below 1.5, 2.0, and 2.5 °C from preindustrial levels, respectively. The IAM simulations in ICA-RUS assume the existence of a policy-maker who presumes climate sensitivity as 3.65 °C, which yields a 66% probability of achieving the policy target by additionally assuming that climate sensitivity follows a normal distribution with a mean of 3.0 and standard deviation of 1.5 (ICA-RUS report 2017).

In addition to T15, T20, and T25, Su et al. (2017) computed a set of future emission pathways for a business-as-usual (BaU) scenario. The T15, T20, T25, or BaU scenarios were each associated with several future emission pathways (temperature pathways) depending on the actual climate sensitivity, which were set to 1.50, 3.00, 3.65, or 4.00 °C. These settings of climate sensitivities were used because the actual climate sensitivity for policy-making is currently unknown; that is, even though policy-makers assume 3.65 °C as the climate sensitivity, future emission pathways (temperature pathways) could differ according to the discrepancy between the actual climate sensitivity and the climate sensitivity assumed by policy-makers. For this reason, four sets of actual climate sensitivities were assumed for each of the policy choices (i.e., T15, T20, T25, and BaU). Although the IAM simulations in the ICA-RUS were also conducted under three Shared Socioeconomic Pathways (SSPs), we focused on SSP2, which is a middle-of-the-road scenario (O’Neil et al. 2014). As a result, our analysis included 16 emission pathways or temperature pathways (i.e., the four temperature goals, including the BaU scenario, multiplied by the four sets of climate sensitivities under SSP2).

**Fig. 2:**
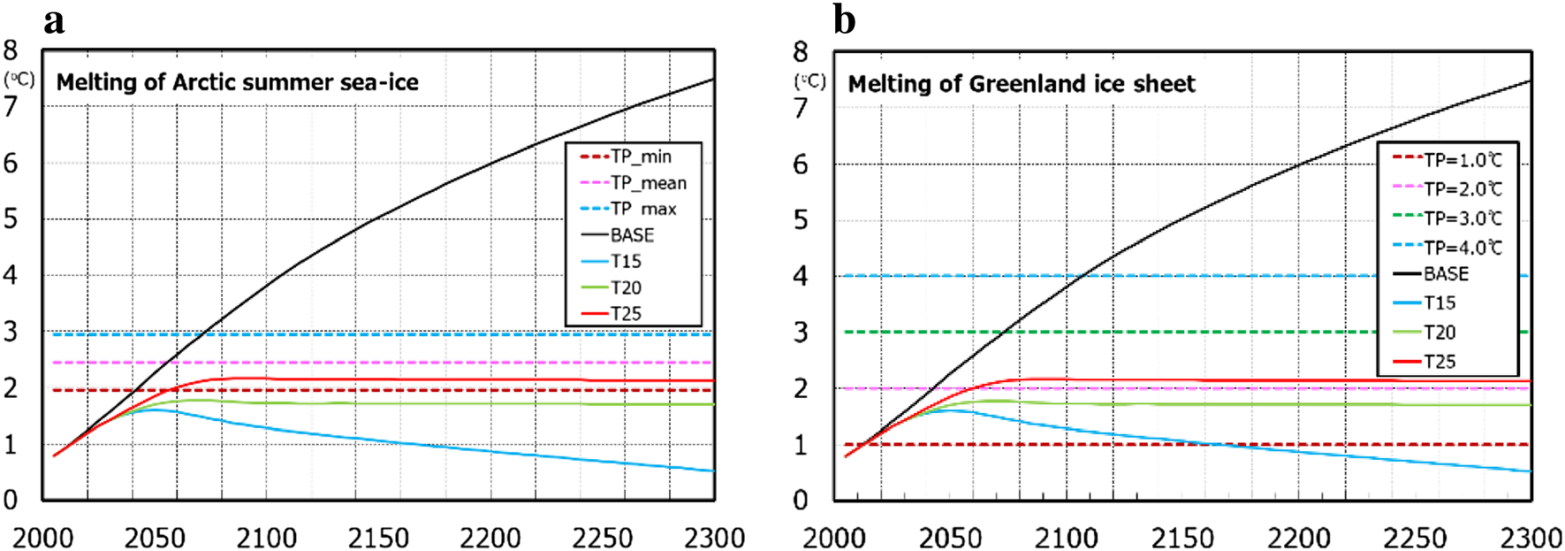
Relationship between global temperature paths of ICA-RUS and threshold temperatures of **a** Arctic summer sea ice and **b** Greenland ice sheet. The temperature paths assume that the climate sensitivity assumed by policy maker is 3.65 °C and the climate sensitivity of actual climate is 3.00 °C. The temperature paths were adjusted so that mean temperature increase during 1986–2005 is 0.61 °C from average of 1850–1900 (IPCC 2013)

Figure 2 shows some of the temperature pathways with threshold ranges of 1.0–4.0 °C for the Greenland ice sheet and 1.95–2.95 °C for Arctic summer sea ice. If the actual climate sensitivity were to differ from the climate sensitivity adopted in a policy, the actual temperature increases at the end of this century would differ from the temperature goal (Su et al. 2015). As such, our framework incorporated this uncertainty in the future temperature increase using the IAM simulations for different climate sensitivities.

**Fig. 3:**
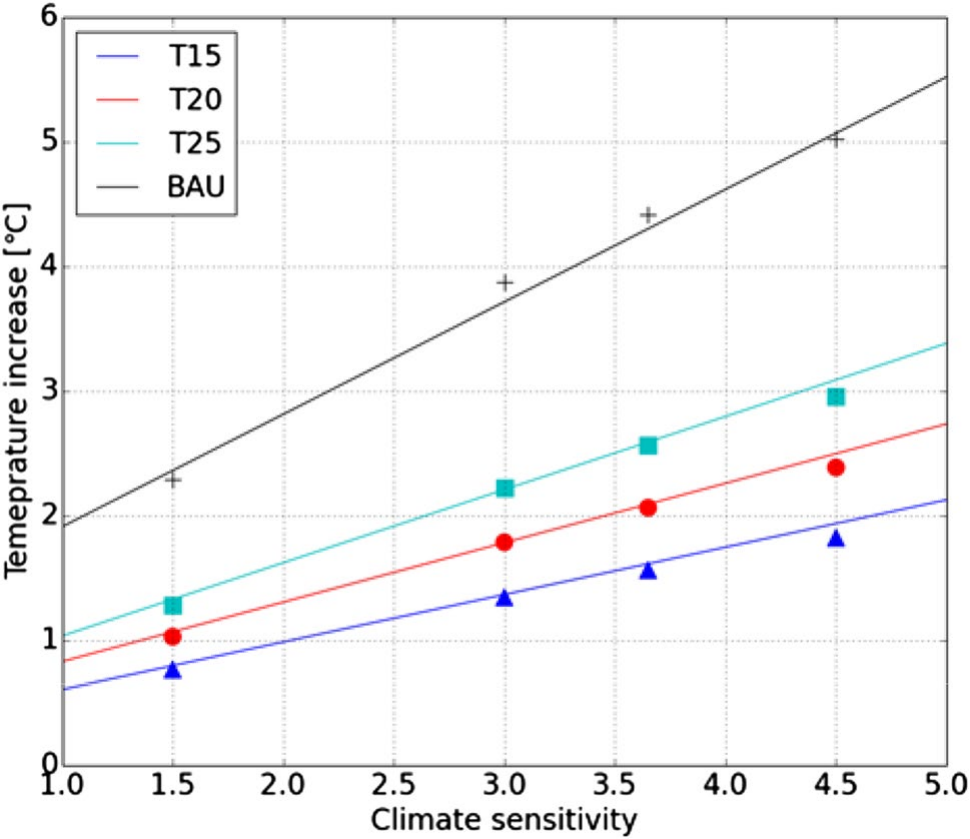
Relationship between the actual climate sensitivities (i.e., actual climate sensitivity = 1.5, 3.0, 3.65, or 4.5 °C) and temperature increase in 2100. The relationship and regression line are shown for the business-as-usual (plus and black line), T15 (triangle and blue line), T20 (circle and red line), and T25 (square and cyan line) scenarios

Using the 16 emission pathways (i.e., 16 temperature pathways) and assumptions of the PDFs of the threshold temperatures, the algorithm in Fig. 1 was executed as follows. First, we estimated the PDFs of the threshold temperature and climate sensitivity (Step 1). The PDF of climate sensitivity was estimated following the assumption used in the ICA-RUS (i.e., climate sensitivity is normally distributed with a mean of 3.0 and standard deviation of 1.5). Then, for each policy choice, we estimated the regression line (Step 2) between the temperature increase in 2100 and actual climate sensitivity (i.e., 1.5, 3.00, 3.65, and 4.5 °C). Scatter plots of the temperature increase in 2100 and actual climate sensitivity suggested that the relationship between the temperature increase in 2100 and actual climate sensitivity could be approximated based on linear regression lines (Fig. 3). Therefore, we used the estimated linear regression lines to convert the PDF of climate sensitivity into the PDF of the temperature increase in 2100 (Step 3).

It should be noted that, although linear response theory of climate sensitivity suggests that the multiplicative (i.e., two) of climate sensitivity should correspond to the same multiplicative (i.e., two) of temperature increase, such multiplicative features do not appear in Fig. 3 (i.e., a twofold increase in the climate sensitivity from 1.5 to 3.0 °C does not correspond to a twofold temperature increase in 2100). The reason for this response feature of temperature increase against climate sensitivity is due to the setting of the target temperature in the IAM. When the climate sensitivity assumed by the policy is in accordance with the climate sensitivity of the actual climate, the temperature increase obtained from the IAM is almost the same as the policy target temperature, as seen at *x* = 3.65 in Fig. 3. However, when the policy assumes a climate sensitivity greater than that of the actual climate, a more stringent effort to reduce emissions is performed in the IAM (Su et al. 2015), and accordingly the temperature increase at 2100 is lower than the policy target temperature. Similarly, when policy assumes a lower climate sensitivity than that of the actual climate, the temperature increase at 2100 exceeds the policy target temperature. In other words, because we use the IAM, which controls emissions according to the policy temperature target and policy-maker assumptions regarding climate sensitivity, the temperature increase at 2100 is strongly controlled by both the temperature target at 2100 and by how much the climate sensitivity of the actual climate departs from the climate sensitivity assumed by the policy. As a result, the temperature increase in 2100 does not show a linear response to increasing of climate sensitivity.

Next, we made a joint PDF from the PDFs of the temperature increase at 2100 and the threshold temperature (Step 4). Finally, we integrated this PDF over the domain where the threshold temperature was lower than the temperature increase at 2100 to obtain probability of exceeding the threshold temperature (Step 5).

### Results and discussion of the probability of exceeding threshold temperatures

**Fig. 4:**
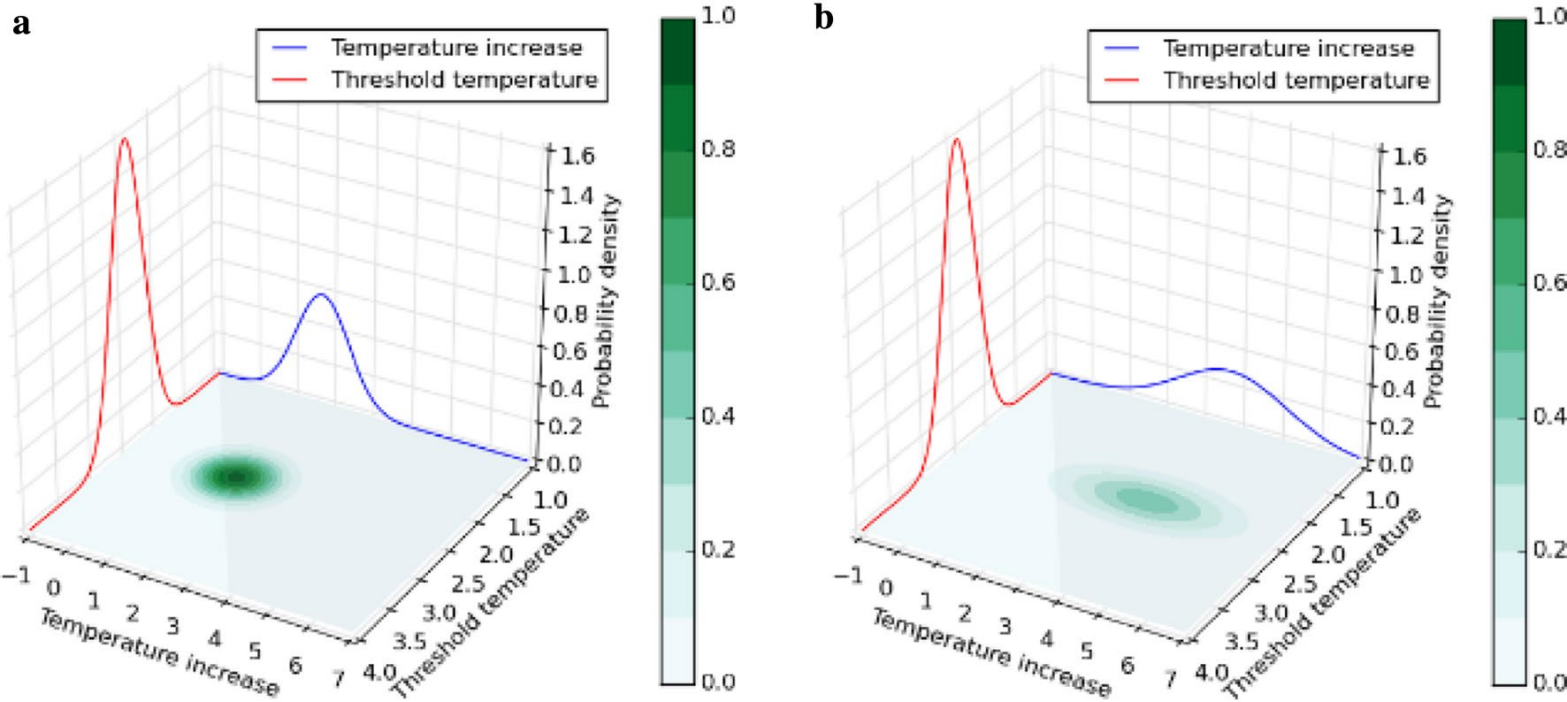
Probability density functions (PDFs) of the threshold temperature for Arctic summer sea ice (red) and temperature increase (blue) at 2100. The PDFs are shown for **a** T20 and **b** business as usual. Joint probability density function (green) between the PDFs is shown on the plain spanned by temperature increase and threshold temperature axis. The domain for integration to obtain the probability of exceeding threshold is colored with gray

**Fig. 5:**
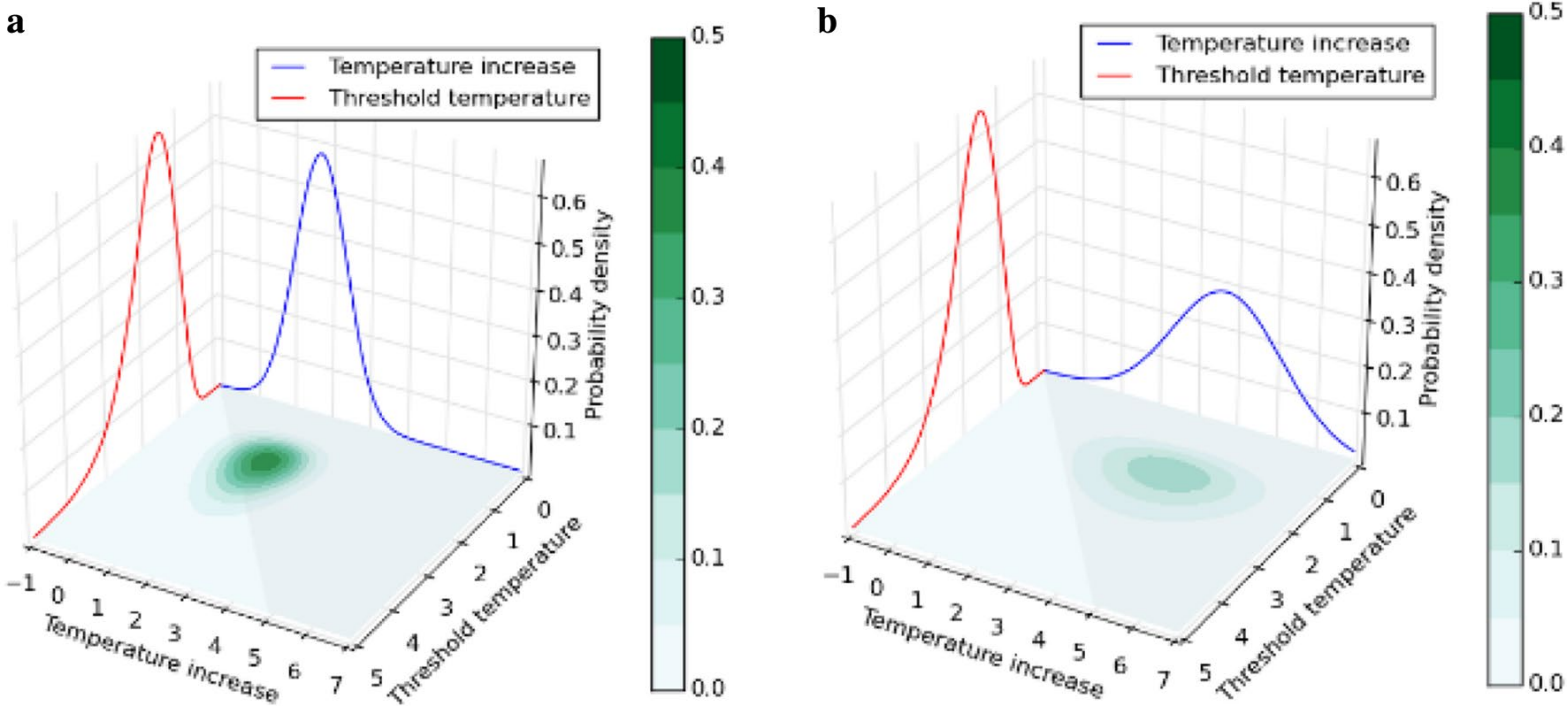
Probability density functions (PDFs) of the threshold temperature for Greenland ice sheet (red) and temperature increase (blue) at 2100. The PDFs are shown for **a** T20 and **b** business as usual. Joint probability density function (green) between the PDFs is shown on the plain spanned by temperature increase and threshold temperature axis. The domain for integration to obtain the probability of exceeding threshold is colored with gray

From the above-described algorithm, we obtained the probabilities of exceeding the threshold temperatures by 2100 (see Step 5 of the algorithm). As examples of how we integrated the joint PDFs to obtain the probabilities of exceeding the threshold, Fig. 4a, b (Arctic summer sea ice under T20 and BaU, respectively) and Fig. 5a, b (Greenland ice sheet under T20 and BaU, respectively) show the joint PDF and the PDFs of the threshold temperature and temperature increase at 2100, respectively. Comparison of Fig. 4a, b (or Fig. 5a, b) shows that BaU has higher probabilities of large temperature increases than T20. Compared with T20, the higher probabilities of larger temperature increases in BaU shift the center of the joint probability density from outside of the integration domain (see Figs. 4a, 5a) to inside of the domain (see Figs. 4b, 5b), resulting in higher probabilities of exceeding the thresholds under BaU. In fact, the cumulative probabilities of exceeding the thresholds, which are obtained by integrating the domain where the temperature increase is greater than the threshold, confirmed that higher target temperatures result in higher probabilities of exceeding the thresholds (see “Appendix 1”).

Table 1 presents the probabilities of exceeding the threshold temperatures for Arctic summer sea ice and the Greenland ice sheet (see Step 5 of the algorithm). The probabilities of exceeding the threshold for the Greenland ice sheet were estimated as 24.8 (13.6)% for T15, 44.5 (26.4)% for T20, 62.5 (39.8)% for T25, and 88.1 (77.6)% for BaU, where the number in parentheses is the probability estimated assuming that the PDF of the threshold temperature follows a uniform distribution. The probability using a uniform distribution was also applied here to investigate the sensitivity of the estimated probability against the choice of probability distribution. A uniform distribution was used for this comparative PDF as an alternative assumption. The probabilities of exceeding the threshold for Arctic summer sea ice were estimated as 2.7 (3.0)% for T15, 16.6 (17.1)% for T20, 37.3 (37.5)% for T25, and 82.4 (82.2)% for BaU. Screen and Williamson (2017) estimated that the probability of a nearly ice-free Arctic sea at the end of summer was less than 1/100,000 for a 1.5 °C increase in temperature. Although our estimation showed a slightly higher probability for T15 than that of Screen and Williamson (2017), both studies indicated that the probability of exceeding the threshold temperature for Arctic summer sea ice would be low under T15.

**Table 1:** Probability (%) of exceeding threshold temperature by 2100

	T15	T20	T25	BaU
Greenland ice sheet	24.8 (13.6)	44.5 (26.4)	60.2 (39.8)	88.1 (77.6)
Arctic summer sea ice	2.7 (3.0)	16.6 (17.1)	37.3 (37.5)	83.4 (82.2)

The numbers in parenthesis represent the probability estimated by assuming that the probability density function of the threshold temperature exhibits a uniform distribution

The range of the uniform distribution is 1.0–4.0 for Greenland ice sheet and 1.95–2.95 for Arctic summer sea ice. These ranges are given as the 95% confidence intervals of the log-normal distribution for Greenland ice sheet and normal distribution for Arctic summer sea ice

The results showed that if the policy target temperature were low, the likelihood of exceeding the threshold for the Greenland ice sheet would be higher than that of Arctic summer sea ice. This was due to our assumption that the lower bound of the threshold for the Greenland ice sheet was lower than that of Arctic summer sea ice. The assumption that the PDF of the Greenland ice sheet was right-skewed also explained the relatively high probability of exceeding the threshold even though the temperature target was low.

However, under a high temperature increase (i.e., BaU), the probability of crossing the threshold for Arctic summer sea ice would rapidly increase, as indicated by a sudden increase in the probability from 37.3% (T2.5) to 82.4% (BaU). This rapid increase in probability arose because the upper bound of the threshold temperature of Arctic summer sea ice was lower than that of the Greenland ice sheet, even though the lower bound of the threshold temperature of Arctic summer sea ice was higher than that of the Greenland ice sheet. Therefore, the probability of exceeding the threshold of Arctic summer sea ice increases rapidly under high temperature increases. However, it should be noted that these trends in the probability of exceeding thresholds strongly depend on the assumptions of the form of the PDF and range of the confidence interval.

Our probabilistic assessment quantitatively showed the variations in the probability of exceeding thresholds according to policy target temperature. By placing assumptions on climate sensitivity and tipping points, this computation suggested that identifying the lower bound of the threshold and narrowing the uncertainty range of the threshold, as well as identifying their PDFs could greatly improve the confidence of the estimated probability of exceeding the threshold temperature.

It should be noted that the probabilities of exceeding thresholds were derived from several assumptions. For instance, we assumed a log-normal distribution for the Greenland ice sheet and a normal distribution for the Arctic summer sea ice, despite the fact that the probability distributions for those thresholds remain unknown. Thereby, a sensitivity analysis of the estimated probabilities was performed using a uniform distribution as the PDF of tipping points. The ranges of uniform distribution for the Greenland ice sheet and Arctic summer sea ice were set as (1.0–4.0) and (1.95–2.95), respectively. The sensitivity analysis (see the numbers in parenthesis in Table 1) suggested that the probabilities varied according to the choice of PDFs. Therefore, it is crucial to keep updating the PDFs and threshold ranges to enhance the reliability of the assessed probabilities of exceeding thresholds. Moreover, further advancement in scientific understanding of climate sensitivity is also vitally important, because previous studies employed various PDFs to fit climate sensitivity (e.g., Hare and Meinshausen 2006; Annan and Hargreaves 2011) and the likely range of climate sensitivity given in AR5 (IPCC 2013) is still wide (i.e., 1.5–4.5).

In addition, the meaning of “exceeding thresholds” should be interpreted carefully. Passing the threshold of the Greenland ice sheet does not mean that it reaches the point of no return; if the temperature reaches only slightly above the threshold and then decreases below the threshold within the transition time, the surface mass balance of the Greenland ice sheet could become positive, although it might not recover to the original state observed before crossing the threshold (Robinson et al. 2012). Passing the threshold of Arctic summer sea ice indicates a nearly ice-free state of Arctic Ocean in September and does not necessarily mean that the climate system of the Arctic Ocean has passed into a different phase; as described in AR5 (IPCC 2013), although sudden loss of Arctic summer sea ice was indicated in some climate projections (Holland et al. 2006; Vavrus et al. 2012; Döscher and Koenigk 2013), those projections are not necessarily associated with the existence of a tipping point to another stable state or irreversible process (Amstrup et al. 2010; Lenton 2012).

## Impact of Greenland ice sheet melting on sea level rise beyond the tipping point

To present the potential impact of crossing the threshold, we focused on the Greenland ice sheet because exceeding its threshold could cause large-scale sea level rise that has a transition time of several hundreds to thousands of years. In fact, the impact of sea level rise associated with global warming on human society has become a matter of concern for coastal areas around the world. With regard to the sea level rise beyond the threshold temperature, most recent studies on sea level rise (Clark et al. 2016; Mengel et al. 2016) and its impacts (Hallegatte et al. 2013; Hinkel et al. 2014; Strauss et al. 2015) have shown only projections to the end of the 21st century or projections that do not model threshold temperatures explicitly. Here, with a focus on limiting temperature scenarios, we describe specific cases of exceeding (T20 and T25) or not exceeding (T15) the threshold temperature. The specific cases were selected based on those which have the highest probability of occurrence under the probabilistic framework outlined in the previous section. Thus, the cases correspond to an actual climate sensitivity of 3.00 °C and threshold temperature of the Greenland ice sheet of 1.6 °C, which are PDF modes for climate sensitivity and threshold temperature. As a result, three emission pathways (i.e., three temperature goals, with SSP2 and an actual climate sensitivity of 3.0 °C) were used for the analysis in this section. To present the global impacts rather than those at a regional scale, only global results that represent the potential sea level rise if the threshold of the Greenland ice sheet melting is exceeded and the degree of impact on society on a millennial timescale are described (see “Appendix 2” for technical details).

**Fig. 6:**
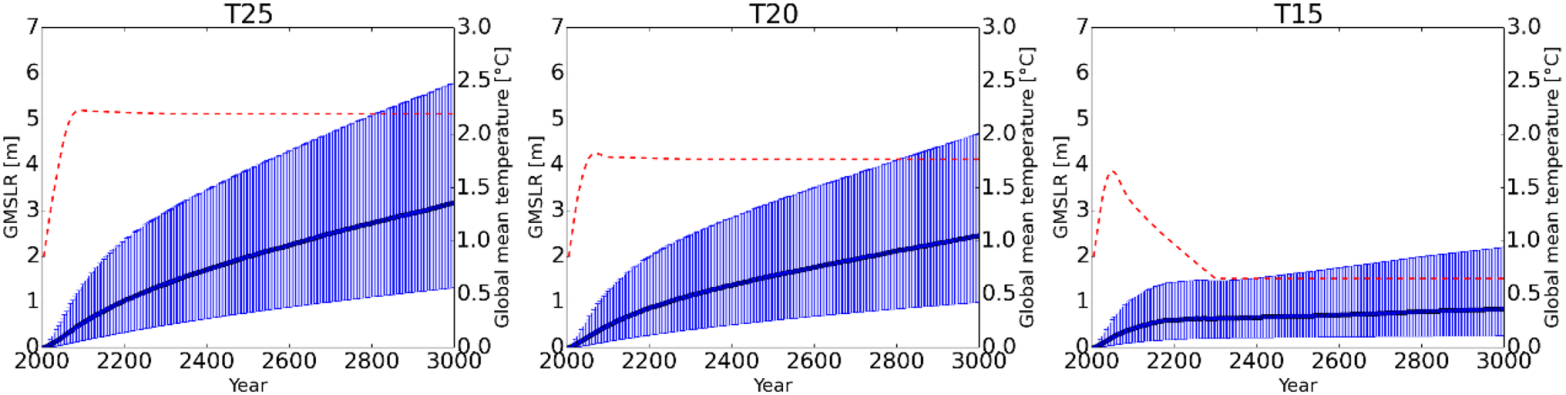
Estimated sea level rise relative to the 2005 level following the three temperature targets of T25, T20, and T15. The blue lines and ranges indicate the median and minimum and maximum ranges of sea level rise relative to 2005, respectively. The red dashed lines indicate the global mean temperature increase relative to preindustrial levels

Figure 6 shows the estimated global sea level rise in the period from 2005 to 3000 under the three temperature goals (T25, T20, and T15). In most of the pathways, the sea level continued to rise even after temperature became constant in 2300 because of the slow response time. Whereas T15 temporarily reaches the Greenland ice sheet threshold of 1.6 °C around the middle of the 21st century, the contribution of the Greenland ice sheet to sea level rise is small because the exceedance of the threshold is slight and occurs for only a short period under T15. For this reason, following discussion regards T15 as the case without exceeding threshold. In the case where the threshold temperature was not exceeded (T15), the sea level rise did not stop, even though the global mean temperature rise showed a decreasing trend, because the attained equilibrium sea level was higher than the current sea level. The maximum range of the estimated sea level rise corresponded to a pessimistic outlook, which assumed the shortest response time and largest expected contribution from all four contributors (see “Appendix 2” for details). Conversely, the minimum range corresponded to an optimistic outlook. Both bounds did not contain the uncertainty of the threshold itself, due to the assumptions.

The estimated sea level rise values associated with T25 and T20, which both exceeded the threshold temperature, were relatively large compared to that of T15, which did not exceed the threshold. The differences in these trends increased over time until the year 3000. Even when the temperature goal was changed from T20 to T15, the differences in the median sea level rise among the ranges was only 0.07 m in 2100, but 0.57 m in 2300 and 1.86 m in 3000. The trends beyond 2100 varied due to the longer response time of the Greenland ice sheet. The global mean temperatures in T25 and T20, which exceeded the threshold of the Greenland ice sheet, were similar, whereas the difference between the global mean temperature in T20 and T15 was relatively large. The uncertainty range of the sea level rise in T15 was smaller than those of T25 and T20 due to the lack of a contribution by the Greenland ice sheet.

**Fig. 7:**
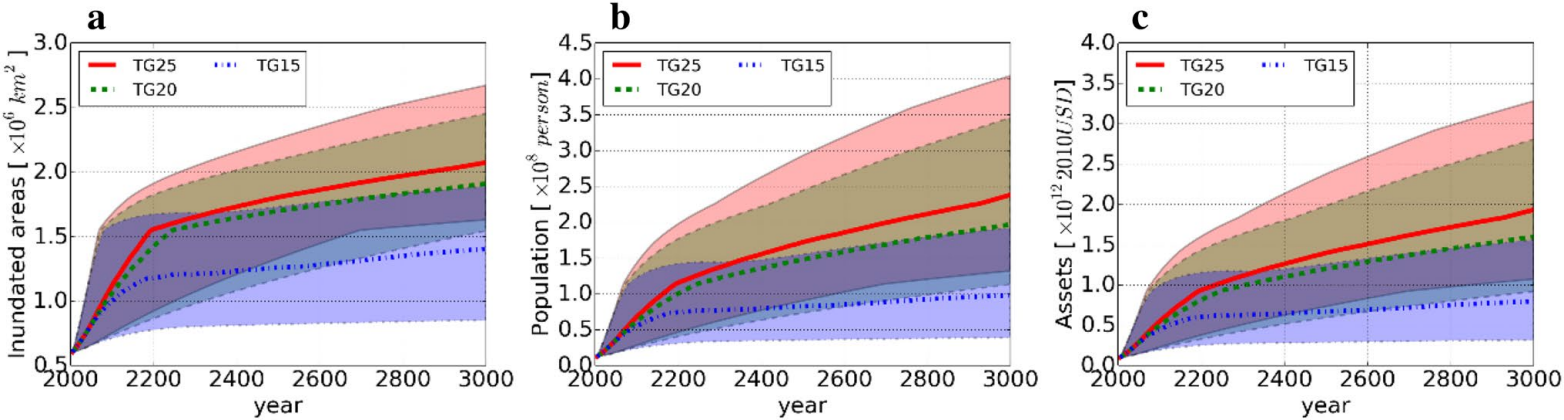
Estimated global total exposure from 2005 to 3000 in terms of **a** inundated area, **b** population, and **c** assets relative to 2005. The red, green and blue line indicates T25, T20, and T15. Each shaded area indicates the estimated minimum and maximum ranges. Greenland threshold of 1.6 °C, the equilibrium climate sensitivity of 3.0 °C, no changes in population and GDP, and no adaptation were assumed

Figure 7 shows the responses of three types of estimated global total exposures: inundated areas (Fig. 7a), population (Fig. 7b), and assets (Fig. 7c). The estimated exposures followed similar trends irrespective of temperature pathway until around 2100. Thereafter, the trends of all exposures in T25 and T20, which continued to exceed the threshold, were relatively similar compared to that of T15, which was considerably lower. This trend became larger over time until the year 3000, because the sea level continued to rise. These increases were relatively steep compared with that of the estimated sea level rise (Fig. 7) because there are broad low-lying areas and concentrated populations in many coastal regions around the world. At a country-scale, countries with either low-lying areas or long coastlines and broad areas of land would suffer more from land loss caused by coastal inundation.

## Concluding remarks

In this study, which had the ultimate aim of incorporating the risks associated with TEs and their thresholds into the development of strategies for global climate risk management, we assessed two major TEs (Arctic summer sea-ice loss and Greenland ice-sheet melting) to determine (i) the probability of passing the thresholds within this century and (ii) the potential impact of the Greenland ice-sheet melting beyond the threshold.

In the first part of this study, we estimated the probability of crossing the thresholds under several policy choices, which indicated how policy choices could alter the probability of crossing thresholds within this century. The estimated probabilities for BaU were significantly larger than those for T25 and T20, whereas the estimated probabilities for T15 were considerably lower. Such information could be used to support decision-making. However, the estimated probability in this study is not definitive, because the derived probability depends on several assumptions, as described in the methodology. Therefore, applying different methods of making assumptions or different analytical approaches would help in evaluating the confidence and robustness of the estimated probabilities. For example, a recent study by Screen and Williamson (2017) used a different estimation approach and found that the probability of achieving a nearly ice-free Arctic sea would be less than 1/100,000 for a 1.5 °C increase and around 1/3 for a 2 °C increase. It should be also noted that the probability of crossing thresholds after 2100 should be researched in greater detail in the future to offer greater support to decision-making.

In the second part of this study, we estimated the global mean sea level rise until the year 3000 using a semi-empirical model assigning three millennial temperature pathways. In addition, we estimated the potential coastal exposure using the estimated sea level.

There was a large gap between the scenarios exceeding the threshold (T25 and T20) and those not exceeding the threshold (T15) in the global total. It should be noted that the results are greatly affected by the assumption that the threshold temperature of the Greenland ice sheet is 1.6 °C. Therefore, the parameters used to describe ice sheets and sea level distribution should be explored further to yield more plausible sea level and exposure estimations on a millennial timescale.

Overall, although our study has presented a framework for probabilistic assessment and added estimates of probabilities of exceeding thresholds while also outlining the potential consequences of exceeding thresholds, enhancing the scientific basis for various TEs and their thresholds through additional research is necessary, because understanding on these parameters is still limited.

To this end, several studies on TEs and their thresholds (e.g., Abe-Ouchi et al. 2013; Yamamoto et al. 2014, 2015; Obase et al. 2017) were successfully conducted for the ICA-RUS. The growth and reduction of Northern Hemisphere ice sheets over a million-year timescale (Abe-Ouchi et al. 2013), and basal melting of the Antarctic ice shelves (Obase et al. 2017) and ocean oxygen depletion due to the decomposition of submarine methane hydrates over timescales of 1,000 years or more (Yamamoto et al. 2014, 2015) could be considered as additional TEs for the next research step. Future policy-related research projects similar to ICA-RUS should continue to promote additional basic studies on TEs and their thresholds.

## Figures and Tables

**Fig. 8:**
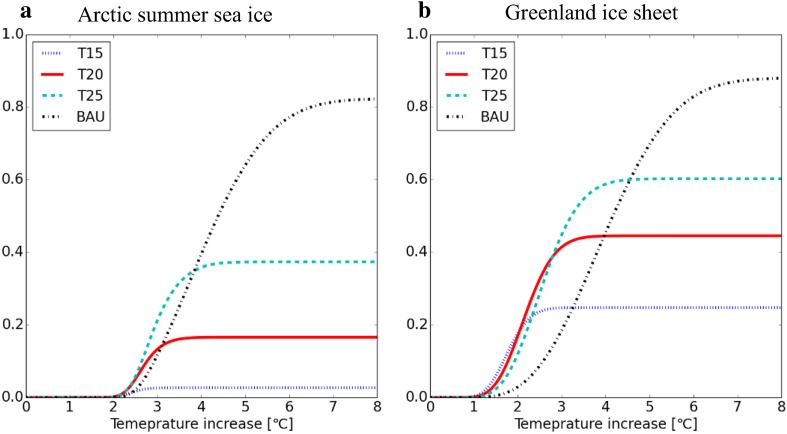
Probabilities on exceeding threshold temperatures as the function of temperature increases in 2100. **a** Arctic summer sea ice and **b** Greenland ice sheet. The cumulative probabilities are computed by integrating the domain of joint density (see Figs. 4a, b, 5a, b) across the temperature increases axis

**Fig. 9:**
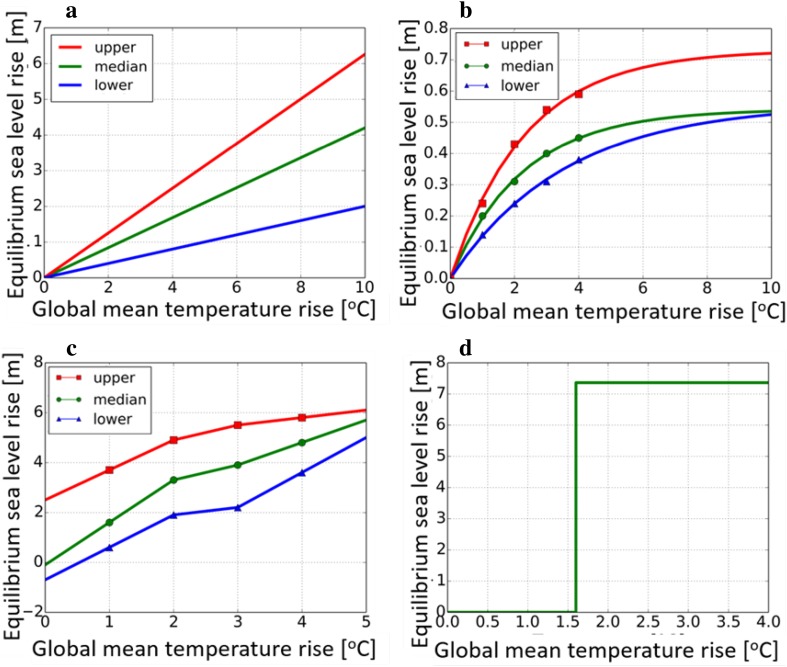
Relationship between global mean temperature above the pre-industrial level and equilibrium SLR of **a** thermal expansion of seawater, **b** glaciers and ice caps, **c** Antarctic Ice Sheet, and **d** Greenland Ice Sheet. Red, green, and blue lines indicate upper bound, median, and lower bound values

## Appendix 1

The probabilities of exceeding threshold temperatures are shown as a function of temperature increase (Fig. 8a for Arctic summer sea ice and Fig. 8b for the Greenland ice sheet). The probability functions were computed by integrating the domain of the joint probability density (see Fig. 4a, b, and Fig. 5a and b) across the temperature increases in 2100. Higher target temperatures were associated with a higher probability of global mean temperature increase, and as a result, the probability of exceeding the thresholds by the end of this century increased because the probability that the threshold temperature would be lower than the temperature increase became higher. Furthermore, T15 had a low probability of achieving high temperature increases (e.g., > 5 °C); therefore, the probability function of T15 reached equilibrium at lower temperature increases. Conversely, the probability function under business as usual (BaU) kept increasing, even at high temperatures, because BaU had a greater probability of high temperature increases.

## Appendix 2

Four global datasets were used to estimate the sea level rise and coastal exposure: global mean temperature, elevation, population, and gross domestic product (GDP) per capita (for details, see Table 2). Our estimation used three emission pathways associated with SSP2 and three global mean emissions pathways by 2100 (T15, T20, and T25) (Su et al. 2017). Actual climate sensitivity was fixed at 3.00 °C. From 2100 to 2300, anthropogenic emissions were maintained roughly at 2100 levels. In T15, T20, and T25, temperature after 2100 was limited to below 1.5, 2.0, and 2.5 °C, respectively (Su et al. 2017). To achieve the 1.5 °C target, a marked reduction in CO_2_ is required, and as a result, T15 reaches negative emissions by 2100. To focus on the millennial-scale effects of exceeding the threshold, this estimation extended the period to the year 3000 with temperature fixed at the level of 2300 (see Fig. 2). This extension was considered to be reasonable because the temperature pathways were similar to the results of the Earth System Models of Intermediate Complexity, with emissions following the representative concentration pathways (Zickfeld et al. 2009).

**Table 2:** Datasets for estimation of coastal exposure

Dataset		Spatial resolution	Period
1. Elevation	GLOBE DEM (Hastings et al. 1999)	30″ × 30″	–
2. Population	Landscan (ORNL 2016)	30″ × 30″	2000
3. GDP	World Bank Data (World Bank 2016)	Each country	2000

The global asset distribution was calculated using the GDP per capita fixed at the year 2000 for each country (World Bank 2016). The asset distribution was calculated by multiplying the population count in grids with the per capita GDP of the country in the same manner as Sato et al. (2014). A fixed population and GDP corresponding to that of 2000 were used for the entire period to highlight the changes between the scenarios that did not, and did, exceed the threshold temperature due to climate change. As such, vertical movements of land, population growth, migration, and socioeconomic development of countries were not considered in this study due to the uncertainty of such projections and calculation costs.

The future global mean sea level rise was estimated using a semi-empirical model (Eq. 1) created by Grinsted et al. (2010) and Mengel et al. (2016) for each sea level contributor. This study divided the contribution to SLR into four contributors: thermal expansion, glaciers and ice caps, Greenland ice sheet, and Antarctic ice sheet. Whereas we considered the threshold for the Greenland ice sheet, the threshold for the Antarctic ice sheet was not considered for the following reasons: Fig. 2d in Leverman et al. (2013) indicates a linear increase in sea level rise with regard to the contribution of the Antarctic ice sheet against temperature increases. In addition, a literature review did not reveal any reliable range of threshold temperature due to the large uncertainties in the predictions of the tipping behavior of the Antarctic ice sheet (i.e., the West Antarctic and East Antarctic ice sheets). This motivated us to use a combination of linear functions, rather than setting a particular threshold for the Antarctic ice sheet.

We defined *S*(*t*) as the time-dependent SLR contribution for each SLR contributor and *S*_eq_(*T*(*t*)) as the long-term sensitivity for the sea level component as a function of the global mean temperature *T*, and *τ* was the response timescale. The short-term rate of SLR can be modeled as a function of global mean temperature as follows:

1 $$\frac{{{\text{d}}S}}{{{\text{d}}t}}=\frac{{{S_{{\text{eq}}}}\left( {T(t)} \right) - S(t)}}{\tau }$$

For the equilibrium sea level of thermal expansion, glaciers and ice caps, and the Antarctic ice sheet, the historical relationship between sea level and temperature derived from 10,000-year runs of six general circulation models, 19 results from runs of two glacier models, and the simulated sensitivity for 5 million years with an ice sheet model (Levermann et al. 2013) were used, respectively (Fig. 9a–c). The given values for the glaciers and Antarctica were the relationships developed at four levels (1, 2, 3, and 4 °C). Fitting, linear interpolation, and extrapolation were used to obtain values other than these points, whereas the values for thermal expansion were the slope values. For the response time for thermal expansion, mountain glaciers and the Antarctic ice sheet to achieve the equilibrium state, the ranges between the upper and lower bounds of the calibrated parameters between the model and observed sea level (Mengel et al. 2016) were applied (Table 3).

**Table 3:** Response time of each contributor. Ranges indicate minimum and maximum ranges for estimated value

Contributor	Response time *τ* (year)
Thermal expansion	81.7–1290.0
Mountain glaciers	89.0–295.0
Antarctic ice sheet	1350.0–2910.0

Although Mengel et al. (2016) used tuple relationships between response time and sea level rise, we assumed that response time and equilibrium sea level rise were independent, so that we could consider the riskiest case of the highest equilibrium state with the shortest response time. Because of this assumption, our estimation provides a more severe case of sea level rise than the estimation in Mengel et al. (2016). However, our worst case scenario of thermal expansion (i.e., combination of the shortest response time with the highest sea level rise of Table S1 in Mengel et al. 2016) would not be completely unrealistic. For example, from Fig. 13.13 and Table 13.8 in AR5 (IPCC 2013), the “high” scenario (> 700 ppm CO_2_) in 2300 could be 1.81 m of sea level rise due to thermal expansion. Figure 12.42 in AR 5 (2013) also indicates that the temperature increase from present-day would be around 3.0 °C in RCP6.0, under which CO_2_ is about 750 ppm after 2100. Considering these figures, and assuming linear relations among temperature increase, response time, and sea level rise, a temperature increase of 1 °C in 100 years would be associated with about 0.6 m of sea level rise. This gives 0.6 and 100 as the tuples of the commitment parameter and calibrated parameter, respectively, which are not significantly different from the worst-case tuples of 0.626 and 81.7. Therefore, so that our simulation included the riskiest situation, we applied the shortest response time with the largest sea level rise given in Table S1 of Mengel et al. (2016).

In addition, the parameters of the Greenland ice sheet were assumed to have a threshold based on the most recently published papers that considered the elevation–temperature feedback associated with surface melting (Robinson et al. 2012; Levermann and Winkelmann 2016). If the global mean temperature were to exceed the threshold of 1.6 °C above the preindustrial level, the Greenland ice sheet would presumably lose its volume irreversibly until an ice-free state equivalent to a 7.36-m SLR was achieved (Fig. 9d). The required time for complete volume loss was defined based on how long, and by how much, the temperature would exceed the threshold. Since the response time of the Greenland ice sheet is a monotonically decreasing function of both the atmospheric lapse rate and the melting sensitivity (Levermann and Winkelmann 2016), the upper and lower limits of the estimates can be computed directly from the observed uncertainty interval of these quantities (see Table 1 of Levermann and Winkelmann 2016). The median estimate for the melting of the Greenland ice sheet varied from more than 2,000 years for a warming of + 1 °C to less than 500 years for a warming of + 5 °C. Here, the time-step was set to 5 years for the global mean emissions pathways.

We estimated the potentially inundated area, population, and assets using the following algorithm. First, the potentially inundated area was estimated for every 1-m increase in sea level, because we used elevation data from the Global Land 1-km Base Elevation (GLOBE) digital elevation model, which has a 1-m vertical resolution and a 30-arc second horizontal resolution. The computation of the potentially inundated area excluded land-locked inland areas that were below sea level. Coastal exposure with each 1-m rise in sea level was obtained from a distribution map of the inundated area, population, and assets. The coastal exposure from 2005 to 3000 was obtained by interpolating each 1-m exposure by the estimated sea level rise. We assumed no adaptation, and thus, the area of potentially inundated land calculated in this study does not fully account for dikes or defenses along coastlines, due to limitations in available data.
